# Evaluating the Potential and Synergetic Effects of Microcins against Multidrug-Resistant *Enterobacteriaceae*

**DOI:** 10.1128/spectrum.02752-21

**Published:** 2022-05-11

**Authors:** Soufiane Telhig, Laila Ben Said, Carmen Torres, Sylvie Rebuffat, Séverine Zirah, Ismail Fliss

**Affiliations:** a Food Science Department, Food and Agriculture Faculty, Laval University, Québec City, Québec, Canada; b Laboratoire Molécules de Communication et Adaptation des Microorganismes, Muséum National d’Histoire Naturelle, Centre National de la Recherche Scientifique, Paris, France; c Department of Food and Agriculture, University of La Rioja, Logrono, Spain; d Institute of Nutrition and Functional Foods, Laval University, Québec City, Québec, Canada; Shenzhen Bay Laboratory

**Keywords:** microcins, bacteriocins, multidrug resistance, antimicrobial activity, RiPPs, synergy

## Abstract

The advent of multidrug-resistant bacteria has hampered the development of new antibiotics, exacerbating their morbidity and mortality. In this context, the gastrointestinal tract reveals a valuable source of novel antimicrobials. Microcins are bacteriocins produced by members of the family *Enterobacteriaceae,* which are endowed with a wide diversity of structures and mechanisms of action, and exert potent antibacterial activity against closely related bacteria. In this study, we investigated the antibacterial activities of four microcins against 54 *Enterobacteriaceae* isolates from three species (Escherichia coli, Klebsiella pneumoniae, and Salmonella enterica). The selected microcins, microcin C (McC, nucleotide peptide), microcin J25 (MccJ25, lasso peptide), microcin B17 (MccB17, linear azol(in)e-containing peptide), and microcin E492 (MccE492, siderophore peptide) carry different post-translational modifications and have distinct mechanisms of action. MICs and minimal bactericidal concentrations (MBC) of the microcins were measured and the efficacy of combinations of the microcins together or with antibiotics was assessed to identify potential synergies. Every isolate showed sensitivity to at least one microcin with MIC values ranging between 0.02 μM and 42.5 μM. Among the microcins tested, McC exhibited the broadest spectrum of inhibition with 46 strains inhibited, closely followed by MccE492 with 38 strains inhibited, while MccJ25 showed the highest activity. In general, microcin activity was observed to be independent of antibiotic resistance profile and strain genus. Of the 42 tested combinations, 20 provided enhanced activity (18 out of 20 being microcin–antibiotic combinations), with two being synergetic.

**IMPORTANCE** With their wide range of structures and mechanisms of action, microcins are shown to exert antibacterial activities against *Enterobacteriaceae* resistant to antibiotics together with synergies with antibiotics and in particular colistin.

## INTRODUCTION

The overuse and misuse of antibiotics in animal and human health generated the emergence of resistance and its spread (1), which are responsible for the antibiotic resistance crisis (2). Recent findings show that human and livestock microbiota have become reservoirs of antimicrobial resistance (AMR) markers (3–5) that can be disseminated to the environment through wastewater treatment or manure (6). This issue is exacerbated by the lack of development of novel antibiotics (7–9). In 2017 the World Health Organization (WHO) warned that ”the world is running out of antibiotics’’ (10). Among the emergent multidrug-resistant (MDR) bacteria, *Enterobacteriaceae* are particularly problematic, with MDR enteropathogens such as Escherichia coli, Klebsiella pneumoniae, and Salmonella strains causing ever frequent outbreaks (11–14). Due to their double membrane and multiple efflux systems (15, 16), *Enterobacteriaceae* infections are notoriously difficult to treat, highlighting their importance as a target for new drug developments. Faced with these challenges, new treatments with reduced risks of resistance emergence and dissemination are needed.

The gastrointestinal tract, which is the seat for multiple microbial interactions, constitutes a valuable source of novel antimicrobials (17, 18), and in particular of bacteriocins, which are antimicrobial peptides produced by bacteria through the ribosomal pathway. Microcins are low molecular weight bacteriocins produced by members of the family *Enterobacteriaceae* (19–21). More specifically, they are peptides below 10 kDa that exhibit potent antimicrobial activities directed against bacteria closely related to the producing strains. Microcins are particularly diverse, many of them being endowed with complex post-translational modifications, making them representatives of ribosomally synthesized and post-translationally modified peptides (RiPPs) (22, 23). Many bacterial species indigenous to livestock and human microbiota produce microcins (20, 24, 25). Furthermore, commercially available probiotics isolated from animal microbiota exhibit the presence of microcin-producing strains (26, 27). Being indigenous to the gut microbiota and exhibiting a narrow spectrum of antibacterial activity, microcins constitute an attractive alternative to antibiotics. While antibiotics can cause significant changes to microbiota compositions and are most often linked to dysbiosis and related disorders (28–31) and can favor AMR dissemination (32), microcins are expected to have a limited impact on the gut microbiota composition and induce a reduced dissemination of resistance, should it emerge. Indeed, other bacteriocins, such as nisin and pediocin PA-1, which both exhibit a narrow spectrum of activity in comparison with antibiotics, have been shown to induce significantly lower changes to the host microbiota (33–36). Given the narrow spectrum of activity of microcins that can vary within the same genus or species, a comprehensive study of their spectra of activity is required to determine which microcin is more appropriate against a given pathogen. Faced with the AMR problem, drug combinations offer a promising reprieve, whereby combining multiple drugs should increase the energetic costs of resistance development (37). As of yet there are no studies exploring the interactions between microcins or between microcins and antibiotics.

**FIG 1:**
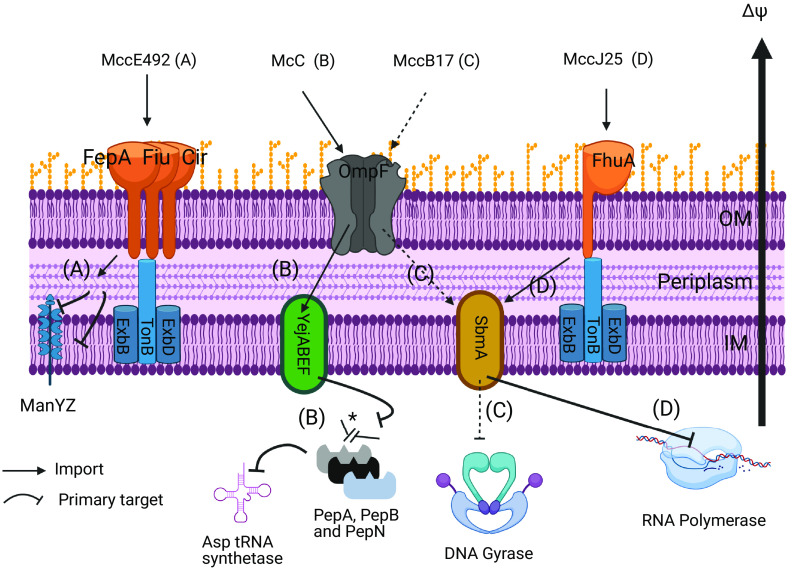
Mechanisms of action of McC, MccJ25, MccB17, and MccE492. MccE492 (A) and MccJ25 (D) gain entry to the periplasm through siderophore receptors FepA/Fiu/Cir (A) or FhuA (D) coupled to the TonB-ExbB-ExbD complex for translocation. McC (B) and MccB17 (C) enter the periplasm through the porin OmpF. McC gains access to the cytoplasm through YejABEF, whereas MccB17 and MccJ25 use SbmA. When in the cytoplasm, MccJ25, MccB17, and McC target RNA polymerase, DNA gyrase, and aspartyl tRNA synthetase, respectively, to kill the cells. For MccE492, it does not enter the cytoplasm but inserts itself into the inner-membrane by stably associating with ManYZ and inducing depolarization of the inner membrane and perturbation of the mannose transport. *, Inside bacteria McC is processed by a deformylase and the peptidases A, B, and N, resulting in the formation of a non-hydrolysable Asp-tRNA mime, thus blocking translation. Figure made with Biorender.

In this study, we explored for the first time the potential of four microcins with specific structures and mechanisms of action, namely, microcins C (McC), J25 (MccJ25), B17 (MccB17), and E492 (MccE492) (Table 1, Fig. 1) (19, 21) to kill, inhibit, or displace a given pathogen using a collection of *Enterobacteriaceae* resistant to antibiotics. *Enterobacteriaceae* from three species, E. coli, K. pneumoniae, and S. enterica, isolated from different origins and exhibiting resistance against different antibiotics were selected for this purpose. The microcins were tested alone or in pairwise microcin-microcin or microcin-antibiotic combinations.

**TABLE 1:** Microcins used in this study

Microcin	Producer	mol wt (Da)	Type of RiPPs	Reference
McC	E. coli	1,177	Nucleotide peptide	72
MccJ25	E. coli	2,107	Lasso peptide	39
MccB17	E. coli	3,093	Linear azol(in)e-containing peptide	41
MccE492	K. pneumoniae	8,781	Siderophore peptide	44

## RESULTS

Four microcins, McC, MccJ25, MccB17, and MccE492, belonging to the RiPP family were selected for their potent and narrow spectrum of activity directed against enteropathogens, their stability to harsh conditions, and their diversity of structures and mechanisms of action (Fig. 1). More precisely, McC is a 7-amino acid nucleotide peptide that is cleaved in susceptible cells to release an aspartyl adenylate mimic that targets aspartyl-tRNA synthetase (38). MccJ25 is a 21-amino acid lasso peptide with an N-terminal macrolactam ring threaded by the C-terminal tail (39), which is imported in susceptible cells through interaction with the membrane proteins FhuA and SbmA and targets RNA polymerase (40). MccB17 belongs to the linear azol(in)e-containing peptide (LAP) family. It contains 43 amino acids, nine of which are converted to thiazole or oxazole rings (41, 42). It is imported in susceptible cells through interaction with the membrane proteins OmpF and SbmA and targets DNA gyrase (43). Finally, MccE492 is a 84-amino acid siderophore-peptide where the C-terminal serine carboxylate is linked to a glycosylated enterobactin derivative (44). It both perturbs the inner membrane permeability and targets the mannose permease (45).

### Production and purification of microcins.

The four microcins were produced heterologously in E. coli and their purification was bio-guided using agar diffusion assays against two reference indicator strains (Table S4). MccJ25, McC, MccB17, and MccE492 were purified at yields of 3.5 mg/L, 9.1 mg/L, 1 mg/L, and 4.0 mg/L of culture, respectively (Table S5).

### Spectrum of activity of the microcins.

The four purified microcins were tested against 54 pathogenic enterobacteria from three species, E. coli, K. pneumoniae, and S. enterica, isolated from different hosts and/or environments: human and animal, animal food, farm indoor air, and wastewater treatment plant (Tables S1-S3). Most of these strains are MDR and their resistance profiles cover a wide panel of antibiotics differing both in structures and mechanisms of action. Out of all the isolates, only three did not exhibit any antibiotic resistance and the rest showed resistances from one to 11 different antibiotics.

**FIG 2:**
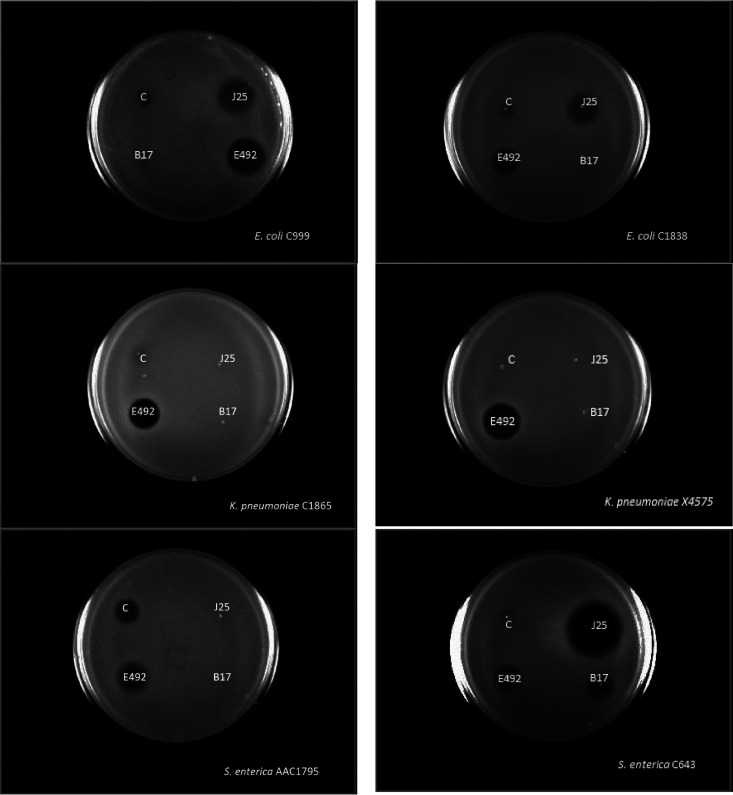
Agar diffusion assays for McC, MccJ25, MccB17, and MccE492 against E. coli, K. pneumoniae, and S. enterica strains. Microcins were deposited at the concentration of 100 μg/mL.

**FIG 3:**
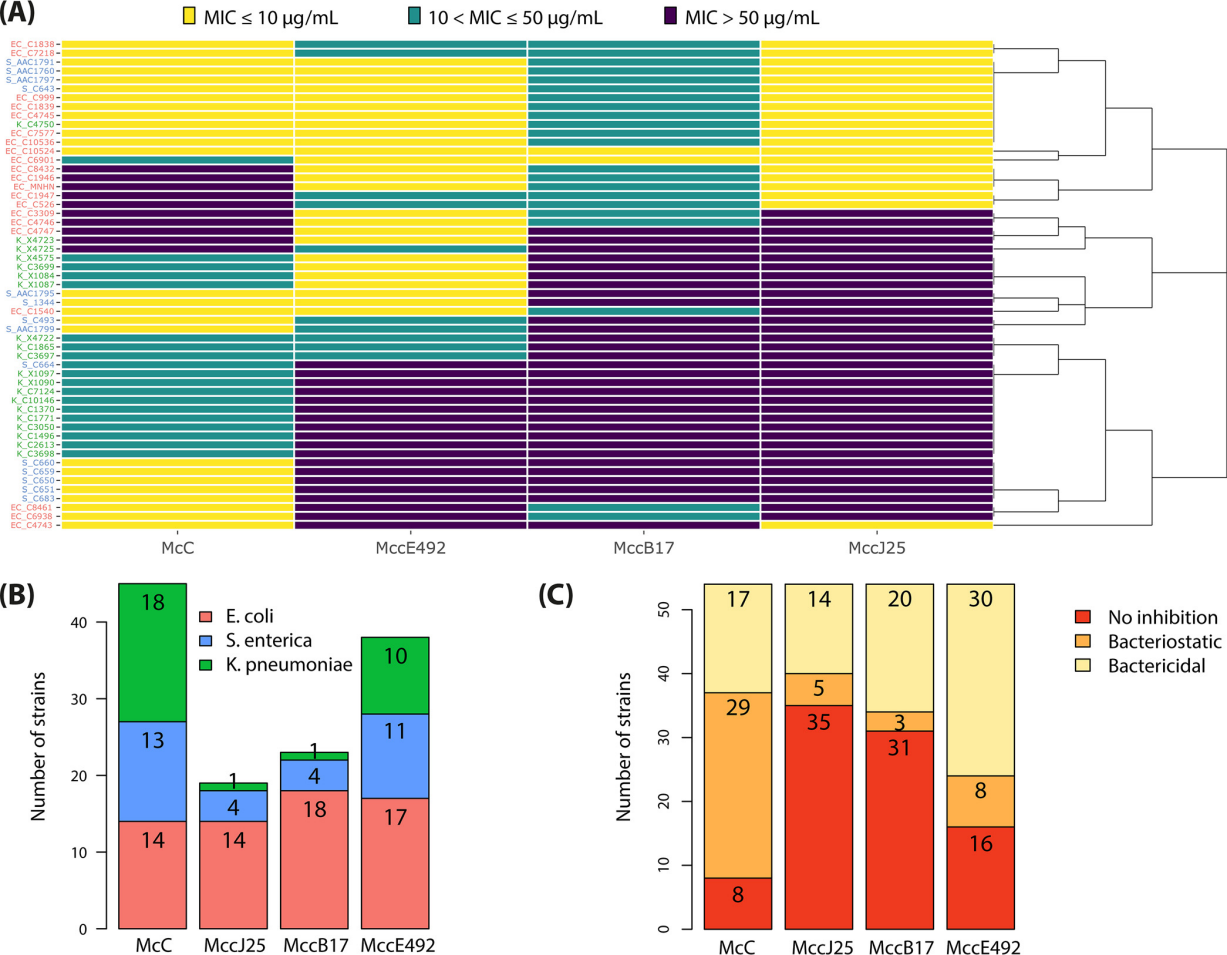
Efficacy of microcins against *Enterobacteriaceae* isolates. McC, MccJ25, MccB17, and MccE492 were tested against a collection of 54 *Enterobacteriaceae* isolates. (A) Heatmap representing the MIC values (in μg/mL). High, medium, and low sensitivity correspond to MIC ≤ 10, 10 < MIC ≤ 50, and MIC > 50 μg/mL, respectively. The corresponding values in μM are provided in Tables S6 to S8. Strains are noted EC for E. coli, K for K. pneumoniae, and S for S. enterica. (B) Susceptibility to microcins per bacterial species. (C) Inhibition type observed per microcin.

The four microcins exhibited heterogeneous spectra of activities, as illustrated by agar diffusion assays (Fig. 2) and MIC measurements (Fig. 3A, Tables S7 to S9). Every isolate showed susceptibility to at least one microcin with MIC values ranging between 0.02 μM and 42.5 μM. The dendrogram constructed from the susceptibility profiles did not reveal a clear clustering per bacterial species. Nevertheless, general trends were observed. The lowest susceptibility corresponded to inhibition by only one microcin, a trend mostly observed for K. pneumoniae, which revealed almost non-susceptible to MccJ25 and MccB17, with the exception of strain K. pneumoniae C4750, susceptible to all four microcins (Table S8). By contrast, E. coli presented both the lowest number of strains susceptible to only one microcin and the highest number of strains susceptible to all four microcins. The number of strains susceptible to each microcin is shown in Fig. 3B. McC revealed the widest spectrum of activity, with 46 strains (85.2%) inhibited within the range of concentrations tested. It was followed by MccE492, which was active against 38 strains (70.4%), then MccB17 with 23 strains (42.6%) and MccJ25 with 19 strains (35.2%). Despite exhibiting the narrowest spectrum of inhibition, MccJ25 presented the lowest recorded MIC (0.02 μM), and thus, the highest efficacy. We assessed the type of inhibition by calculating the ratio R between the MIC and MBC (Fig. 3C). When *R* > 4, the activity of a given antimicrobial compound is considered bacteriostatic, while it is considered bactericidal for *R* ≤ 4 (46). Microcin inhibition varies between bacteriostatic and bactericidal depending on the tested strains. MccJ25, MccB17, and MccE492 appear mainly bactericidal while McC, which displays the widest spectrum of activity, appears mainly bacteriostatic.

**FIG 4:**
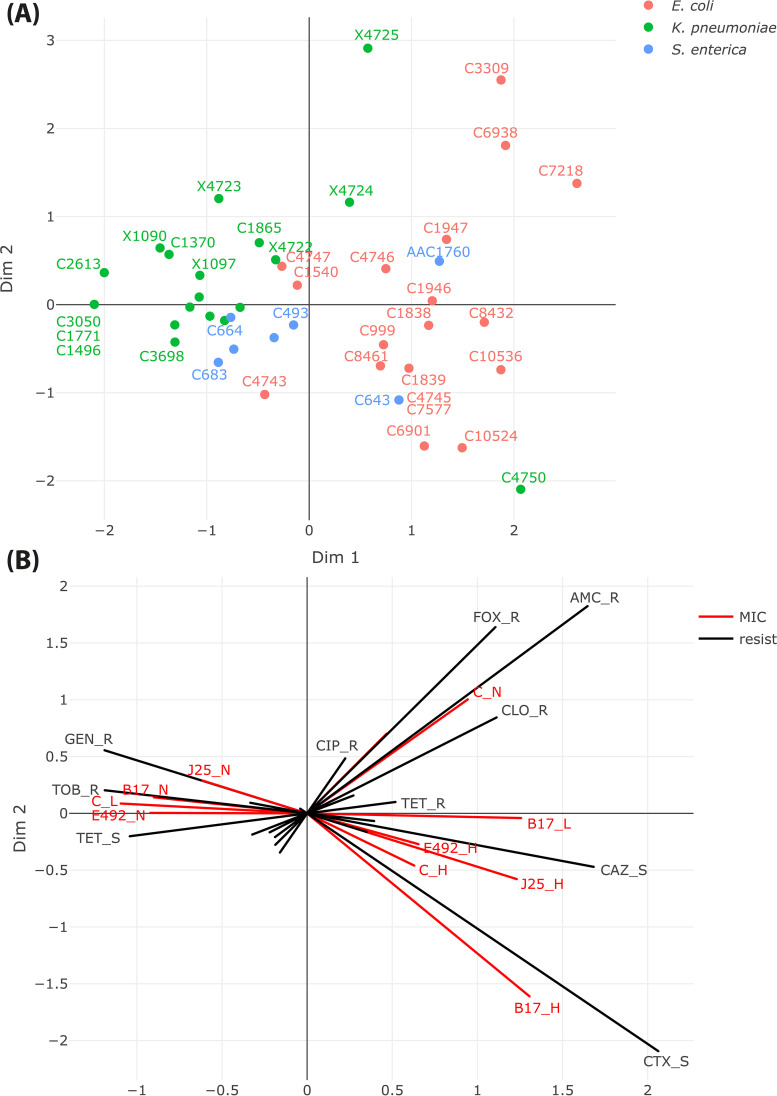
MFA correlations identified between microcin and antibiotic susceptibilities. (A) Representation of the strains in the first two dimensions, colored per species. (B) Representation of the susceptibility categories in the first two dimensions. In red: susceptibility to microcins (H, high, MIC ≤ 10 μg/mL, L, low, 10 < MIC ≤ 50 μg/mL, N, no activity up to 50 μg/mL); in black: susceptibility to antibiotics (R, resistant, S, susceptible).

To assess the relationships between microcin susceptibility and antibiotic resistance profiles, we performed multifactorial analysis (MFA) based on categorized susceptibility to microcins and antibiotics (Fig. 4). MFA revealed a clustering for K. pneumoniae (except for isolate C4750) and E. coli, while S. enterica showed a more heterogeneous distribution (Fig. 4A). The strains that showed most susceptible to the microcins were projected on the bottom-right panel, while the strains less susceptible to microcins (weakly susceptible to MccC), and especially all K. pneumoniae, clustered on the upper left panel. Representation of the microcin and antibiotic susceptibility categories in the first two components of the multicomponent analysis (MCA) (Fig. 4B) suggested several associations between resistances to a specific microcin and a specific antibiotic (namely, gentamicin/MccJ25, tobramycin/MccB17, and amoxicillin-clavulanic acid/McC). The MFA analysis shows that resistance phenotypes toward MccJ25, gentamicin, and tobramycin pointed in the same direction. However, only the relationship between gentamicin and MccJ25 resistances was confirmed by Chi-square test for independence.

### Microcin-microcin and microcin-antibiotic combinations.

**FIG 5:**
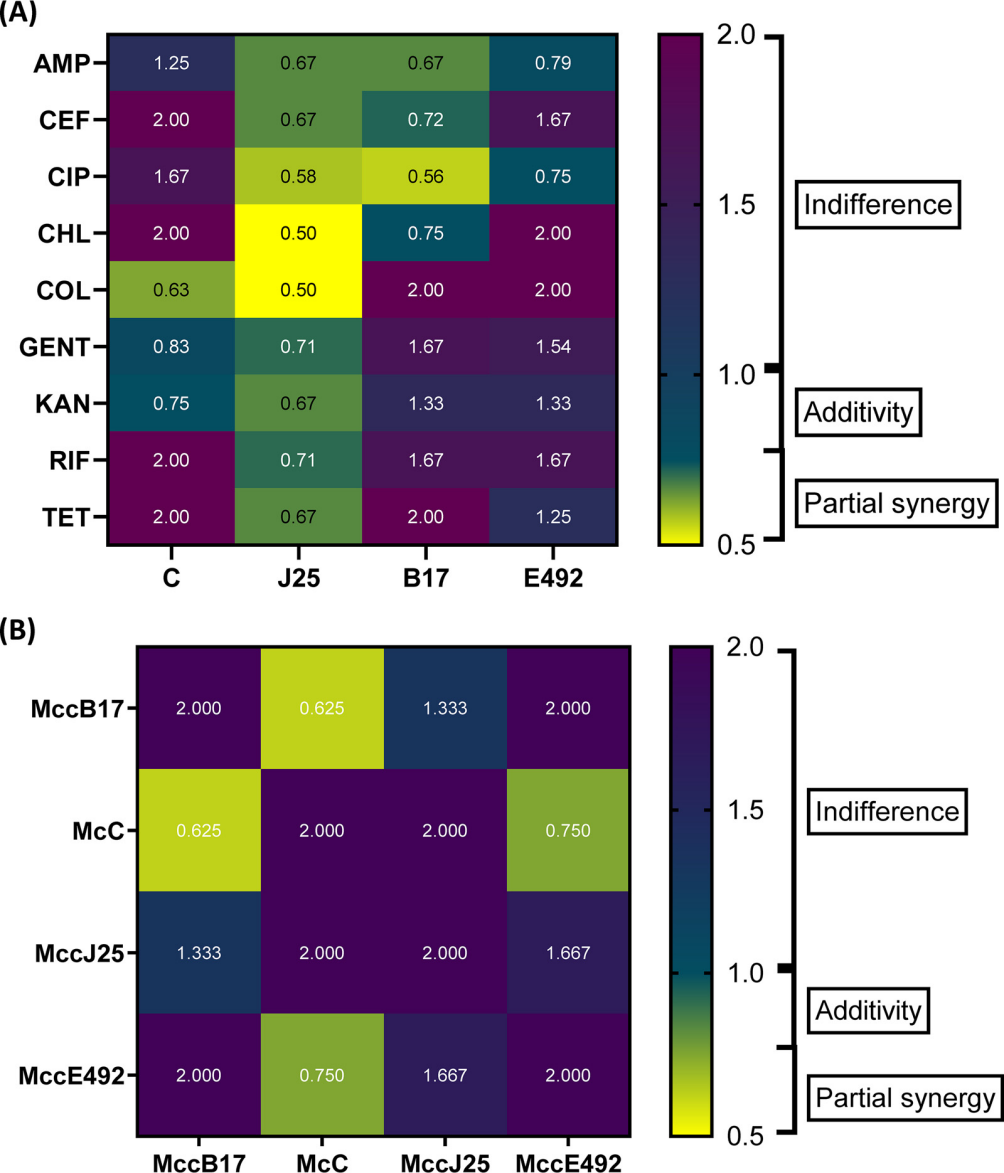
FIC values measured for all consortia tested in the study against E. coli and Salmonella indicator strains. (A) shows the FIC values for the antibiotic microcin-consortia. (B) shows the FIC values for the microcin-microcin consortia. FIC ≤ 0.5: synergy, 0.5 < FIC ≤ 0.75: partial synergy, 0.75 < FIC < 1: additivity and 1 ≤ FIC ≤ 2 = indifference.

We further looked at the possibility of combining microcins in different consortia either with each other or with different antibiotics presenting diverse mechanisms of action. The MICs of the antibiotics and microcins alone against the indicator strains were first determined (Table S6). Then, 42 consortia were tested and the fractional inhibitory concentration (FIC) index, indicative of the combination effect (47), was determined (Fig. 5). Out of all the tested combinations, 22 were observed to be indifferent (52.4%), two were additive (4.8%), 16 were partially synergetic (38.1%), and two were synergetic (4.8%) (Fig. 6). Interestingly, no antagonistic effect was detected.

All the combinations between MccJ25 and antibiotics exhibited interactions within the partial synergy range, whereas McC combined with antibiotics produced two interactions within the partial synergy range with colistin (FIC = 0.63) and kanamycin (FIC = 0.75) (Fig. 5). The interactions between microcins and antibiotics were much more synergetic than pairwise microcin-microcin interactions. Out of the 20 positive interactions (FIC < 1), pairwise microcin-microcin combinations only accounted for two (10%) with MccB17/McC and MccE492/McC. Indeed, the only synergetic effects were observed for MccJ25/colistin and MccJ25/chloramphenicol combinations. MccB17 accounted for five partially synergetic in total. McC exhibited three partially synergetic and one additive effects in total, while MccE492 showed one additive and two partially synergetic interactions (Fig. 5). The most potent beneficial interactions between microcins and antibiotics are listed in Table 2.

**TABLE 2:** Most potent synergetic interactions^*a*^

Microcin	MICc/MIC	Antibiotic	MICc/MIC	FIC index
MccJ25	1/4	Chloramphenicol	1/4	0.50
MccJ25	1/4	Colistin	1/4	0.50
MccJ25	1/2	Ciprofloxacin	1/16 to 1/32	0.58
MccB17	1/2	Ciprofloxacin	1/16	0.56
McC	1/2	Colistin	1/16	0.63

a MICc, MIC of the compound in the combination; MIC, MIC of the compound alone. MICc/MIC is the ratio between the resulting MIC of an antimicrobial agent within the consortium and the MIC of the same antimicrobial by itself.

## DISCUSSION

The objective of this study was to evaluate the potential of microcins as a treatment against *Enterobacteriaceae* notorious for their capacity to develop resistances to conventional antibiotics (15, 16) and or persist within the host (48, 49). Despite reviews discussing the prospects of using microcins as novel therapeutics, due to their high specificity, potent antibacterial activity and reduced collateral damage to the host’s microbiome (19, 21, 22, 50), there have been few comprehensive studies on this point.

Hence, we first investigated whether four microcins (McC, MccJ25, MccB17, and MccE492) can exert bacterial inhibition against a variety of *Enterobacteriaceae*, both MDR and non-MDR. In total, 54 natural isolates were tested, belonging to the species E. coli, K. pneumoniae, and S. enterica, all considered urgent and/or serious threats by the Centre for Disease Control and Prevention (CDC). None of the studied *Enterobacteriaceae* were non susceptible to all microcins within the range of tested concentration (up to 50 μg/mL). However, there was a high variability in the effectiveness of the different microcins to inhibit the growth of bacteria. The lowest recorded MIC values were 0.03 μM, 0.1 μM, 1 μM and 2 μM for MccJ25, MccE492, McC, and MccB17, respectively. Concomitantly, McC recorded the widest spectrum out of all studied microcins, with 85.2% of strains inhibited within the range of concentrations used, followed by MccE492 with 70.4% of strains inhibited. According to the data, the most efficient microcins were MccE492 and MccJ25, albeit with the narrowest spectrum for MccJ25 (35.2%). In the case of MccE492, these observations are in line with previous studies, whereby siderophore microcins including MccE492 were found to confer a significant fitness advantage (26). Moreover, D’Onofrio et al. (51) have shown that adding exogenous siderophores to synthetic media can promote the growth of previously uncultured bacteria, via iron acquisition. Their importance is increased in the context of a host, where iron availability is reduced, suggesting MccE492, and siderophore microcins by extension, are promising therapeutics.

Looking at the type of inhibition effectuated by microcins it was observed to be variable throughout the different strains, changing between bacteriostatic and bactericidal. Indeed, the highest variation in the type of inhibition activity is observed for McC and MccJ25. Both microcins were recorded to have two different mechanisms of action, with one acting in the cytoplasm and the second at the level of the inner membrane but at much higher concentrations, close to mM (52–55). Although the two mechanisms are assumed to be independent, in reference to the concept that has been proposed for conventional antibiotics (56), it could also be considered that for both microcins the main mechanism of action could result, at least for a part, in deleterious changes in the inner membrane, leading consecutively to a bactericidal effect. Both alternatives could explain the variety observed within their activity. Moreover, it cannot be discarded neither that reduction of microcin uptake at the outer membrane, essentially via the involved receptor or porin, reduces the microcin concentration in the cytoplasm, which thus cannot reach the level required for a bactericidal activity. Furthermore, the activities of the tested microcins seemed independent of the species and antibiotic resistance profiles of the pathogens. Given that the receptors and targets of these microcins are well characterized, a comprehensive genomic study is needed to shed more light on any potential association between susceptibility to a given microcin and the antibiotic resistance profile or the species of a given strain.

Comparing microcin susceptibilities and antibiotic resistance profiles of the strains revealed a correlation between MccJ25 and gentamicin resistances. Although the mechanisms of action of these two pairs of antimicrobial molecules are different, this result suggests that cross-resistance may occur between microcins and conventional antibiotics. It must be noted that the resistance to antibiotics of the collection of *Enterobacteriaceae* was measured phenotypically. Furthermore, despite the detection of antibiotic resistance genes coding for acquired resistance enzymes, it remains unclear whether these resistances are due to acquired AMR genes or if mutational events on the targets of the antibiotics could also be implicated. Hence, concerning the statistical relevance of the correlation between gentamicin and MccJ25 resistance, an in-depth study of the mechanisms of aminoglycoside resistance should be performed in these isolates, mostly those related to mutations in the antibiotic target, to detect potential microcin-antibiotic resistance interactions. Moreover, a larger collection of gentamicin-susceptible and gentamicin-resistant isolates could be analyzed in the future.

Secondly, we investigated whether it was possible to enhance microcin activity by combining them between each other or with a collection of antibiotics. This is a standard method to circumvent antibiotic resistance and in keeping with the comprehensive aim of this study, we chose antibiotics covering a wide range of mechanisms (membrane degradation, inhibition of cell wall biosynthesis or of protein synthesis by targeting the 30S and 50S ribosome, etc.). There were no observations of any antagonistic effects, yet no observation of a highly significant synergy (FIC < 0.5). The most promising consortia were observed between microcins and antibiotics. MccJ25 presented the most enhanced interactions, with the best FIC indexes measured for combinations with chloramphenicol and colistin. Colistin interacts with the bacterial cytoplasmic membrane changing its permeability (57), which could explain its ability to enhance MccJ25 activity, presumably by increasing the microcin uptake, which would then rely on both import through FhuA and SbmA and membrane permeabilization. At high concentrations, MccJ25 has also been observed to cause membrane perturbations and disruption of the cytoplasmic membrane gradient (55), which could also contribute to the synergy between colistin and this microcin. MccJ25 and chloramphenicol both inhibit protein synthesis but using different mechanisms, i.e., by blocking transcription through binding to RNA polymerase for MccJ25 (54) and by targeting translation for chloramphenicol, which reversibly binds to the 50S ribosomal L16 protein (58). These combined effects could explain the synergetic effect between MccJ25 and chloramphenicol. Tetracycline, kanamycin, and gentamicin, all 30S inhibitors (59, 60), present a lower FIC index when combined to MccJ25. This suggests that combinations of MccJ25 with 50S inhibitors are more beneficial than with 30S inhibitors.

Given that there is a finite number of entry pathways into a target cell, there was overlap in the mechanisms of the tested compounds. For instance, MccJ25 and rifampicin share the FhuA and SbmA receptors they use for uptake, and RNA polymerase as their cytosolic target, yet no antagonism was observed between them. According to Mathavan et al. (61), MccJ25 occupies a location within FhuA similar to that of ferrichrome, its natural ligand. Moreover, ferrichrome and the antibiotics rifampicin and albomycin were also shown to occupy similar sites within FhuA (62). Furthermore, while MccJ25 targets the β’ RNA polymerase sub-unit (63), rifampicin targets the β sub-unit (64). These different mechanisms at the level of the RNA polymerase interaction coupled with the lack of tight structural specificity of FhuA for its ligands could explain the lack of antagonism between MccJ25 and rifampicin. On the other hand, SbmA also presents the ability to accommodate and transport various substrates including antimicrobial peptides such as MccB17 (65) and proline-rich AMPs (66). Indeed, the use of SbmA is shared by both MccB17 and ciprofloxacin on one hand and MccJ25 and ciprofloxacin on the other. Both consortia recorded similarly partially synergetic effects at 0.56 and 0.59, respectively. The lack of antagonism despite sharing SbmA as a receptor has also been recorded for ciprofloxacin and rifampicin (67, 68), suggesting that both SbmA and FhuA structural specificity is not a limiting factor when designing drug combinations. It could be hypothesized that the use of these consortia, would increase the potential of cross-resistance emergence. Nevertheless, combining ciprofloxacin and rifampicin was shown to reduce the frequency of resistance emergence in comparison with ciprofloxacin alone, which was attributed to rifampicin killing any ciprofloxacin resistant subpopulation (67).

No significant enhancing effects were recorded by combining MccE492 with antibiotics. MccE492 activity relies on simultaneously disrupting mannose transport and inner membrane pore formation through binding to the mannose permease (45). This suggests that microcins with a cytoplasmic target are more prone to exert synergic activities with antibiotics.

Combining two by two, the microcins did not reveal much synergetic effects, with the exception of McC and MccB17. This could be due to the narrow spectrum of activity of microcins. It must be noted that our results show different spectra of activity of the microcins, coupled with the lack of antagonism when combining different microcins. It could thus be beneficial to use microcin consortia, not necessarily for synergetic effects, but to cover a wider spectrum of pathogens. It must be noted, however, that the frequency of natural resistance emergence to microcins is still poorly studied. Yet, both the lack of antagonism and enhancing effects between microcin and antibiotics shown in this study appear promising, especially in the case of colistin, due to the toxicity of the latter.

To summarize, the microcins tested were effective against the collection of *Enterobacteriaceae* isolates within the range of concentrations tested. McC exhibited the widest range of activity, whereas MccJ25 accounted for the lowest MIC values. Furthermore, both McC and MccJ25 activities presented the highest variation in type of inhibition. Finally, all the tested combinations exhibited significantly varying FIC indexes, with microcin-antibiotics combination having the most synergetic effects observed.

## MATERIALS AND METHODS

### Bacterial strains and plasmids.

The E. coli, K. pneumoniae, and S. enterica isolates along with their serotypes and resistance profiles to antibiotics are listed in Tables S1, S2, and S3, respectively. These strains were obtained from the collection of the University of La Rioja (Logroño, Spain) and from Agriculture Canada’s pathogen collection and their phenotypes and genotypes of resistance were known from previous studies. The E. coli strains used for heterologous production of microcins together with their microcin-encoding plasmids, and the indicator strains used for susceptibility assays are listed in Table S4. Two reference indicator strains were also used, E. coli ATCC 25922 and S. enterica subsp. *enterica* serovar Newport ATCC 6962 (later termed *S.* Newport ATCC 6962).

### Production and purification of microcins.

The microcin producing strains were cultured in LB medium or in M63 minimal medium (for 1 L, 3 g KH_2_PO_4_, 7 g K_2_HPO_4_, 2 g (NH_4_)_2_SO_4_, 1 g Casamino Acids) supplemented with glucose (2 g/L), thiamine (1 mg/L), and MgSO_4_ (0.2 g/L). Ampicillin (Amp) or chloramphenicol (Chl) were added as selection factor when appropriate, at 50 μg/mL and 34 μg/mL, respectively. For all microcins, an overnight culture of the producing E. coli strain was grown at 37°C and 200 rpm and used at 1% to inoculate 500 mL of supplemented M63 medium, for an overnight culture in 2 L erlemneyers at 37°C and 200 rpm. The cultures were centrifuged at 12,000 rpm and 4°C for 20 min. For McC, MccJ25, and MccE492, the culture supernatants were collected. For MccB17, the pellet was collected and suspended in 25 mL acetic acid 100 mM, EDTA 1 mM, and heated at 100°C under shaking at 80 rpm. The resulting suspension was then centrifuged at 4,250 *g* and 4°C for 20 min for collection of the clear supernatant. The culture supernatants of strains producing McC, MccE492, and MccJ25 and pellet extract of strain producing MccB17 were treated by solid phase extraction on a Sep-Pak C18 35 cc (Waters) for MccB17, McC, and MccJ25, or on a Sep-Pak C8 35 cc (Waters) for MccE492. In all cases, the cartridges were conditioned with methanol, acetonitrile (ACN), and 0.1% trifluoroacetic acid (TFA) (A1, for McC, MccB17 and MccJ25) or formic acid (FA) in milliQ water (A2, for MccE492), successively. After loading the supernatants, the cartridges were washed with A1 or A2 and then eluted with A1 or A2 together with increasing the amount of ACN. McC, MccB17, MccJ25, and MccE492 were eluted with 10%, 25%, 30%, and 40% ACN, respectively. The modified form of MccE492, in which the C-terminal Ser residue is connected to three N-(2,3-dihydroxybenzoyl) units through a β-d-glucose moiety (44), was purified. The SPE fractions were then concentrated and submitted to reverse phase high performance liquid chromatography (RP-HPLC), using mobile phase A1 (for McC, MccB17 and MccJ25) or A2 (for MccE492) together with ACN. McC, MccB17, and MccJ25 were purified from SPE fractions on a Mandel Shimadzu 2D HPLC system, using a C18 Phenomenex column (Luna 10 μm, 250 mm × 21.10 mm) at 6 mL/min, and a gradient from 0% to 50% ACN in 20 min, and to 100% ACN in 10 min. MccE492 was purified on a biocompatible RSLC HPG-3400RS chromatographic system (Thermo Fisher Scientific), on a Luna C18(2), 250 × 4.6 mm, 100 Å, 5 μm column (Phenomenex) at 1 mL/min, using a gradient from 32% to 42% ACN in 22 min, and to 100% ACN in 1 min.

Peptide purification was monitored upon testing the antibacterial activity of the collected fractions against two reference indicator strains (Table S4). Purity of the collected microcins was checked by analytical HPLC and LC-MS (Fig. S1) and determined as ≥ 95%. Quantification of the microcins was obtained using BCA and Lowry assays.

### Liquid chromatography–mass spectrometry.

The purified peptides were analyzed by liquid chromatography–mass spectrometry (LC-MS) on a high-resolution electrospray—quadrupole—time of flight (ESI-Q-TOF) instrument, using either a 1290 Infinity II UPLC (chromatography system connected to a hybrid ion mobility Q-TOF instrument (6560, Agilent), for MccJ25, McC and MccB17, or an Ultimate 3000-RSLC system (Thermo Fisher Scientific) connected to a Maxis II ETD ESI-Q-TOF instrument (Bruker Daltonics), for MccE492. For the former LC-MS system, the separation was achieved on a Poroshell 120 EC-C18 column (2.1 × 100 mm, 2.7 μm, Agilent) at a flow rate of 400 μL/min, using an A2/ACN gradient from 10% to 100% ACN over 15 min. For the latter system, the separation was achieved on a Polar Advantage II Acclaim column (2.2 μm, 120 Å, 2.1 × 100 mm, Thermo Fisher Scientific) at a flow rate of 300 μL/min, using an A2/ACN gradient from 10% to 100% ACN over 15 min. The MS detection was performed in positive mode.

### Antibacterial assays. (i) Agar diffusion assays.

The tested strains were cultured overnight in LB medium at 37°C and 200 rpm before being inoculated at 1% into soft agar LB medium (0.75%). Wells were dug out and 80 μL of microcin were added in each well and the plates were incubated at 37°C overnight.

### (ii) Measurement of minimal inhibitory and bactericidal concentrations (MIC and MBC).

MIC determination was carried out using the broth microdilution assay in 96-well plates and following the Clinical and Laboratory Standards Institute (CLSI) guidelines. Two-fold serial dilutions of antibiotic were obtained starting from stock solutions at 100 μg/mL. Plates were incubated at 37°C, and growth was measured as absorbance at 600 nm over a period of 18 h. The MIC was determined as the lowest concentration that completely inhibited the bacterial growth. The MBC was determined by inoculating a MH agar surface with 10 μL from wells showing complete inhibition and incubating for 24 h at 37°C.

Antibiotic stock solutions were prepared following the CLSI guidelines and aliquoted in MilliQ water for –20°C storage. Microcins were aliquoted in MilliQ water at 200 μg/mL and stored at –20°C.

### (iii) Measurement of FIC indexes.

Interdependent effects analysis for all the tested combinations was performed in triplicates against the two indicator strains E. coli ATCC 25922 and *S.* Newport ATCC 6962 (Tables S4 to S6), using the microdilution checkerboard method following the CLSI guidelines. The FIC index was interpreted as follows (47): synergetic effect FIC ≤ 0.5, partial synergy 0.5 < FIC ≤ 0.75, additivity 0.75 < FIC < 1, neutral 1 ≤ FIC ≤ 4, and antagonism FIC > 4. For the MccJ25, MccB17, and MccE492 combinations with antibiotics, E. coli ATCC 25922 was used as indicator strain. For McC and antibiotics combinations, *S*. Newport ATCC 6962 was used. Concerning the pairwise microcin-microcin combinations, *S*. Newport ATCC 6962 strain was used for McC/MccJ25 and McC/MccE492 while E. coli ATCC 25922 was used for the other combinations.

### Statistical analysis.

All statistical analyses were performed in R software version 4.1.1. Chi-square test for independence was used to test for independence between antibiotic and microcin susceptibility. Multivariate factorial analysis (MFA) was performed using FactoMineR (69) and graphical representations were constructed using factoextra (70) and plotly (71) packages. Two groups of variables were considered: (i) microcin susceptibility, categorized into high (MIC ≤ 10 μg/mL), low (10 < MIC ≤ 50 μg/mL) and no (MIC > 50 μg/mL) activity and (ii) antibiotic susceptibility, categorized into sensible (S) and resistant (R). The bacterial species was considered as supplementary variable.
