# Supplementary material for: Evaluating the Potential and Synergetic Effects of Microcins against Multidrug-Resistant Enterobacteriaceae
Source: Microbiol Spectr. 2022 May 11;10(3):e02752-21. doi: 10.1128/spectrum.02752-21 (PMC9241698; doi:10.1128/spectrum.02752-21)
Supplement: SUPPLEMENTAL FILE 1 — Supplemental material. Download spectrum.02752-21-s001.pdf, PDF file, 1.2 MB [file spectrum.02752-21-s001.pdf]

Evaluating the potential and the synergetic effects of microcins against multi-drug resistant *Enterobacteriaceae*

Soufiane Telhig [1,2], Laila Ben Said [1], Carmen Torres[3], Sylvie Rebuffat [2], Séverine Zirah [2], Ismail Fliss [1,4 *]*

[1] Food science department, Food and agriculture faculty, Laval University, Quebec, Quebec, Canada

[2] Laboratoire Molécules de Communication et Adaptation des Microorganismes, Muséum National d'Histoire Naturelle, Centre National de la Recherche Scientifique, Paris, France

[3] Department of Food and Agriculture, University of La Rioja, Logrono, Spain

[4] Institute of Nutrition and Functional Foods, Laval University, Quebec, Quebec, Canada

Supporting information

Strain	ESBL + other beta-lactamases	Phenotype of R	Genotype R (for non beta-lactams)	Origin	Country
C526	CTX-M-9, TEM-1	AMP/CTX/CAZ/STR/TET/SUL/SXT/NAL	*tetA, sul1, sul2, dfrA16, aadA2*	Clinical faecal sample[a]	Spain
C999	CTX-M15, TEM-1, OXA-1	AMP/CTX/CAZ/CIP/TET/SUL/SXT/TOB/KAN/STR	*tetA, sul1, aac(6´)-ibcr, dfrA17, aadA5*	clinical urine sample[a]	Spain
C1540	CTX-M-15	AMP/CTX/CAZ/NAL/CIP/TET/SUL/SXT/GEN/TOB/STR	*tetA, aac(6´)-Ib-cr, dfrA17, aadA5*	clinical faecal sample[a]	Spain
C1838	SHV-12	AMP/CTX/CAZ/NAL/TET/SUL/CLO/STR	*tetA, aadA2, cmlA, sul3*	chicken food[b]	Spain
C1839	CTX-M-14a	AMP/CTX/CAZ/NAL/CIP/TET/STR	*tetA*	chicken food[b]	Spain
C1946	CTX-M14a	AMP/CTX/CAZ/NAL/CIP/TET/SUL/SXT/STR/KAN	*tetA, aadA, sul1, sul3, aph(3´)-Ia*	chicken food[b]	Spain
C1947	SHV-12, TEM-1	AMP/CTX/CAZ/NAL/CIP/TET/SUL/CLO/STR	*tetB, aadA2, cmlA, sul3*	chicken food[b]	Spain
C4743	TEM-52c	AMP/CTX/CAZ/KAN/STR		chicken food[b]	Spain
C4745	SHV-12, CTX-M-1	AMP/CTX/CAZ/NAL/TET/SUL	*tetA, sul2*	chicken food[b]	Spain
C4747	CTX-M-9	AMP/CTX/CAZ/NAL/TET/SUL/SXT/GEN/STR	*tetA, sul1, aadA2, dfrA16, aa(3´)-I,*	chicken food[b]	Spain
C3309	CMY-2	AMP/CTX/CAZ/AMC/FOX/CIP/STR/CLO/SUL/SXT/TET	*int2, cmlA*	pork	Mexico
C4746	SHV-12	AMP/CTX/CAZ/CIP/STR/SUL/SXT/TET	*int2, tetB, sul2, dfrA1, sat2, aadA1ST57*	chicken meat	Spain
C6901	CTX-M-1	AMP/CTX/CAZ/NAL/ STR/ SUL/ SXT/ TET	*int2, sul2, tetA, dfrA12-sat2-aadA1*	chicken meat	Tunisia
C7218	CMY-2	AMP/CTX/CAZ/FOX/AMC/STR/CLO/TOB/SXT/TET	*int2, tetA*	turtle, faeces	Mexico
C7577		AMP/CTX/CAZ/NAL/STR/TMP/TET	*int2*	roe deer, faecal sample	Spain
C8432	CTX-M-3	AMP/CTX/CIP/NAL/STR/SUL/SXT/TET	*int2*	chicken, faecal sample	Spain
C8461		AMP/CTX/NAL/STR/SUL/SXT/TET	*int2*	chicken faecal sample	Spain
C10524	CTX-M-14	AMP/CTX/CAZ/TET/CIP/STR	*mcr-1*	air pig farm	Spain
C10536	CTX-M-14	AMP/CTX/CAZ/TET/CLO/SXT		air pig farm	Spain
C6938	CMY-2	AMP/CTX/CAZ/AMC/FOX/NAL/CIP/SXT/TET/AMK/CLO	*dfrA14, aadA1, sul1, tetA*	chicken, faeces	unknown

Table S1: List of natural *E. coli* isolates used in microcin activity assays.

Phenotypic and genotypic data of these isolates were obtained in previous studies, [a] human sample, [b] chicken farm sample. ESBL: extended spectrum beta-lactamase

Antibiotic abbreviations: amoxicillin-clavulanic acid (AMC), amikacin (AMK), ampicillin (AMP), cefotaxime (CTX), ceftazidime (CAZ), chloramphenicol (CLO), ciprofloxacin (CIP), cefoxitin (FOX), gentamicin (GEN), kanamycin (KAN), nalidixic acid (NAL), tetracycline (TET), tobramycin (TOB), sulfamethoxazole (SUL), trimethoprim-sulfamethoxazole (SXT).

Strain	ESBL + other β-lactamases	Phenotype of Resistance	Genotype R (for non beta-lactams)	Origin	Country
C1370	CTX-M-15, SHV-11, OXA-1	AMP/CTX/CAZ/FOX/TOB/KAN/GEN/TET/CLO	*aac(6´)-IbcrA, oqxAB, aac(3)II, aph(3´)-Ia*	clinical faecal sample[a]	Spain
C1771	CTX-M-15, SHV-11, OXA-1	AMP/CTX/CAZ/FOX/TOB/KAN/GEN	*aac(6´)-IbcrA, oqxAB, qnrS1, aac(3)II, aph(3´)-Ia*	clinical sample[a]	Spain
C1865	CTX-M-15, SHV-28, OXA-1	AMP/CTX/CAZ/FOX/TOB/KAN/TET/CLO/SUL/SXT	*aac(6´)-IbcrA, oqxAB, sul1, dfrA12, aadA2*	clinical sample[a]	Spain
C3050	CTX-M-15, SHV-11, OXA-1	AMP/CTX/CAZ/FOX/TOB/KAN/GEN	*aac(6´)-IbcrA, oqxAB, qnrS1, aac(3)II, aph(3´)-Ia*	clinical sample[a]	Spain
C1496	CTX-M-15, SHV-11, OXA-1	AMP/CTX/CAZ/FOX/TOB/KAN/GEN	*aac(6´)-IbcrA, oqxAB, qnrS1, aac(3)II, aph(3´)-Ia*	clinical sample[a]	Spain
C2613	CTX-M-15, SHV-11, OXA-1	AMP/CTX/CAZ/FOX/CIP/TOB/KAN/GEN/FOS	*aac(6´)-IbcrA, oqxAB, qnrS1, aac(3)II, aph(3´)-Ia*	clinical sample[a]	Spain
C3698	CTX-M-3	AMP/CTX/CAZ		WWTP[b]	Algeria
C3697	CTX-M-15, TEM-1	AMP/CTX/CAZ/FOX/TET/SUL	*qnrB1*	WWTP[b]	Algeria
C3699	CTX-M-15, SHV-11, OXA-1	AMP/CTX/CAZ/FOX/GEN/SUL	*qnrB1, aac(6´)-Ibcr, catB4*	WWTP[b]	Algeria
C4750	SHV-12, TEM-1	TET/SUL/SXT/STR	*dfrA1, aadA,1, tetA, sul1, sul2,*	chicken food[b]	Spain
X4722	CTX-M-15, SHV-28, TEM-1	AMP/CTX/CAZ/FOX/ TET/NAL/CIP/SXT/STR	*tetA*	faecal sample, dog	Spain
X4575	CTX-M-15, SHV-1	AMP/CTX/CAZ/CIP		faecal sample, dog	Spain
X4724	CTX-M-15, SHV-28	AMP/CTX/CAZ/FOX/TET/CIP/SXT	*int1, tetA*	faecal sample, dog	Spain
X4723	SHV1, TEM-1	AMP/CTX/CAZ/FOX/TET/CIP/GEN/STR	*int1, tetA*	faecal sample, dog	Spain
X4725	SHV-28, TEM-1	AMP/CTX/CAZ/FOX/TET/CIP/SXT/GEN/STR		faecal sample, dog	Spain
X1084	KPC, SHV-1	AMP/CTX/CAZ/FOX/IMP/CIP/SXT/STR/TOB		clinical isolate[a]	Portugal
X1087	KPC, SHV-1	AMP/CTX/CAZ/FOX/IMP/CIP/SXT		clinical isolate[a]	Portugal
C7124	CTX-M-15, TEM-1b, SHV-28	AMP/CTX/CAZ/FOX/TET/SXT/NAL/TOB		Hospital environment	unknown
X1090	CTX-M-15, SHV-27, KPC	AMP/CTX/CAZ/FOX/CIP/SXT/TET/GEN/STR/TOB		clinical isolate[a]	Portugal
X1097	CTX-M-15, SHV-11	AMP/CTX/CAZ/FOX/CIP/SXT/TET/TOB		clinical isolate[a]	Portugal

Table S2: List of natural *K. pneumoniae* isolates used for microcin activity assays.

Phenotypic and genotypic data of these isolates were obtained in previous studies, [a] human sample, [b] wastewater treatment plant (WWTP); ESBL: extended spectrum beta-lactamase.

Antibiotic abbreviations: amoxicillin-clavulanic acid (AMC), amikacin (AMK), ampicillin (AMP), cefotaxime (CTX), ceftazidime (CAZ), chloramphenicol (CLO), ciprofloxacin (CIP), cefoxitin (FOX), gentamicin (GEN), kanamycin (KAN), nalidixic acid (NAL), tetracycline (TET), tobramycin (TOB), sulfamethoxazole (SUL), trimethoprim-sulfamethoxazole (SXT).

Strain	Serovar	ESBL + other β-lactamases	Resistance phenotype (non-beta lactams)	Origin	Country
C643	Rissen	SHV-12, TEM-1	AMP/CTX/CAZ/TET/STR/SUL	Pig faecal sample[a]	Spain
C659	Virchow	CTX-M-9, TEM-1b	AMP/CTX/CAZ/STR/SUL/SXT/NAL	Chicken faecal sample[a]	Spain
C660	Virchow	CTX-M-9, TEM-1b	AMP/CTX/CAZ/STR/SUL/SXT/NAL	Chicken faecal sample[a]	Spain
C664	Enteritidis	CTX-M-9, TEM-1b	AMP/CTX/CAZ/TET/STR/SUL/SXT/NAL	Chicken faecal sample[a]	Spain
C650	Virchow	CTX-M-9, TEM-1b	AMP/CTX/CAZ/STR/SUL/SXT/NAL	faecal sample[b]	Spain
C651	Virchow	CTX-M-9, TEM-1b	AMP/CTX/CAZ/TET/STR/SUL/SXT/NAL	faecal sample[b]	Spain
C683	Virchow	CTX-M-10, TEM-1b	AMP/CTX/CAZ/SUL/NAL	faecal sample[b]	Spain
C493	Livingstone	SHV-2	AMP/CTX/CAZ/TET/STR/SUL/	faecal sample[b]	Spain
AAC 1799	Typhimurium	Unknown	None	Unknown	Canada
AAC 1797	Enteririditis	Unknown	None	Unknown	Canada
AAC 1760	Heidelberg	Unknown	AMP/AMC/CTX/FOX/CAZ	Unknown	Canada
AAC 1791	Kentucky	Unknown	None	Unknown	Canada
AAC 1795	Unknown	Unknown	None	Unknown	Canada
1344	Unknown	Unknown	None	Unknown	France

Table S3: List of natural *Salmonella enterica subsp. enterica* isolates used for microcin activity assays.

Phenotypic and genotypic data of these isolates were obtained in previous studies. [a] sample from slaughterhouse, [b] sample from human with acute gastroenteritis.

Antibiotic abbreviations: amoxicillin-clavulanic acid (AMC), amikacin (AMK), ampicillin (AMP), cefotaxime (CTX), ceftazidime (CAZ), chloramphenicol (CLO), ciprofloxacin (CIP), cefoxitin (FOX), gentamicin (GEN), kanamycin (KAN), nalidixic acid (NAL), tetracycline (TET), tobramycin (TOB), sulfamethoxazole (SUL), trimethoprim-sulfamethoxazole (SXT).

Strain	Plasmid	Antibiotic cassette	Produced microcin	Reference
E. coli TG1[a]	pBR325	Ampicillin	McC	[1]
E. coli MC4100[a]	pTUC202	Chloramphenicol	MccJ25	[2]
E. coli DH5α[a]	pUC19mccB17	Ampicillin	MccB17	[3]
E. coli MC4100[a]	pJAM229	Ampicillin	MccE492	[4]
E. coli ATCC 25922[b]	None	None	None	ATCC [5]
S. enterica Newport ATCC 6962[b]	None	None	None	ATCC [6]

Table S4: List of laboratory strains used in the study. [a] recombinant *E. coli* strains used for microcin production. [b] indicator strains used for microcin activity control and FIC index experiments.

Microcin		[Total protein] (µg/mL)	[Purified peptide] (mg)	Activity AU/mL	Specific activity AU/mg
McC	Spt	219.45	109.7	32	145.8
	SPE	199	39.8	128	643.2
	HPLC	912.63	9.1	1024	1122
MccJ25	Spt	171.8	171.8	32	149
	SPE	357.2	71.4	256	717
	HPLC	353.5	3.5	2048	5793
MccB17	Spt	99.45	49.7	16	321.8
	SPE	100	12.5	64	640
	HPLC	212.18	1.1	512	2413
MccE492	HPLC	NA	4	2048	NA

Table S5: Production yields for McC, MccJ25 and MccB17. Activities were measured in AUs using the microtitration method in a 96 well plate of the appropriate production phase. Spt is the active supernatant obtained after culture in complemented M63 medium. SPE is the active fraction containing the microcin after SPE. HPLC corresponds to the collected microcin peak in RP-HPLC. NA: not acquired

Antimicrobial compounds	Target strains			
	E. coli ATCC 25922		*S.* Newport ATCC 6962	
Antibiotics				
	µg/mL	µM	µg/mL	µM
Ampicillin	1.56	4.46	1.56	4.46
Cefotaxime	6.75	14.8	6.75	14.8
Ciprofloxacin	0.39	1.20	0.19	0.60
Chloramphenicol	0.01	0.03	<0.01	<0.03
Colistin	0.8	0.69	0.8	0.69
Gentamicin	0.39	0.82	0.39	0.82
Kanamycin	3.12	6.44	1.56	3.22
Rifampicin	0.80	0.97	0.39	0.47
Tetracycline	4.00	9.00	4.00	9.00
Microcins				
	µg/mL	µM	µg/mL	µM
McC	3.12	2.66	3.12	2.66
MccJ25	0.78	0.37	0.06	0.03
MccB17	3.12	1.01	3.12	1.01
MccE492	1.56	0.18	1.56	0.18

Table S6: MIC values (µg/mL and µM) for the antibiotics and microcins used in the FIC index experiments against *E. coli* and *Salmonella* indicator strains.

Strain	Microcin							
	McC		MccJ25		MccB17		MccE492	
	µg/mL	µM	µg/mL	µM	µg/mL	µM	µg/mL	µM
C526	>50ND	>42.5ND	0.09^{B}	0.04^{B}	25.00^{B}	8.08^{B}	18.75^{b}	2.15^{b}
C999	3.12^{B}	2.66^{B}	1.95^{B}	0.93^{B}	18.75^{B}	6.06^{B}	1.17^{B}	0.13^{B}
C1540	3.12^{B}	2.66^{B}	>50ND	>23.7ND	31.25^{B}	10.10^{B}	6.25^{B}	0.72^{B}
C1838	3.90^{B}	3.32^{B}	0.11^{B}	0.05^{B}	25.00^{B}	8.08^{B}	18.75^{B}	2.15^{B}
C1839	1.56^{B}	1.33^{B}	3.17^{B}	1.51^{B}	18.75^{B}	6.06^{B}	9.37^{B}	1.08^{B}
C1946	>50ND	>42.5ND	0,34^{B}	0.16^{B}	18.75^{B}	6.06^{B}	1.56^{b}	0.18^{b}
C1947	>50ND	>42.5ND	0.12^{B}	0.06^{B}	18.75^{B}	6.06^{B}	18.75^{B}	2.15^{B}
C4743	2.34^{B}	1.99^{B}	7.03^{B}	3.34^{B}	>50ND	>16.2ND	>50ND	>5.7ND
C4745	1.56^{B}	1.33^{B}	0.09^{B}	0.04^{B}	12.50^{B}	4.04^{B}	4.69^{B}	0.54^{B}
C4747	>50ND	>42.5ND	>50ND	>23.7ND	>50ND	>16.2ND	2.34^{B}	0.27^{B}
C3309	>50ND	>42.5ND	>50ND	>23.7ND	>50ND	>16.2ND	2.34^{B}	0.27^{B}
C4746	>50ND	>42.5ND	>50ND	>23.7ND	>50ND	>16.2ND	9.37^{B}	1.08^{B}
C6901	12.50^{b}	10.62^{b}	0.43^{b}	0.21^{b}	6.25^{B}	2.02^{B}	1.17^{B}	0.13^{B}
C7218	6.25^{B}	5.31^{B}	0.14^{B}	0.07^{B}	18.75^{B}	6.06^{B}	37.50^{B}	4.30^{B}
C7577	1.56^{B}	1.33^{B}	0.24^{B}	0.11^{B}	12.50^{B}	4.04^{B}	6.25^{B}	0.72^{B}
C8432	>50ND	>42.5ND	0.44^{B}	0.21^{B}	18.75^{B}	6.06^{B}	4.69^{B}	0.54^{B}
C8461	3.12^{B}	2.66^{B}	>50ND	>23.7ND	37.50^{B}	12.12^{B}	>50ND	>5.7ND
C10524	3.12^{B}	2.66^{B}	0.05^{B}	0.03^{B}	6.25^{B}	2.02^{B}	4.68^{B}	0.54^{B}
C10536	2.73^{b}	2.32^{b}	0.14^{B}	0.07^{B}	12.50^{B}	4.04^{B}	1.56^{B}	0.18^{B}
C6938	4.68^{b}	3.98^{b}	>50ND	>23.7ND	37.50^{B}	12.12^{B}	>50ND	>5.7ND

Table S7: Microcins McC, MccJ25, MccB17 and MccE492 MIC mean values in µg/mL and µM against *E. coli* natural isolates. The MBC legend is ^{b} for bacteriostatic effect, ^{B} for bactericidal effect and ND for an immeasurable MBC, due to no inhibition within the range of microcin concentrations used.

Strain	Microcin							
	McC		MccJ25		MccB17		MccE492	
	µg/mL	µM	µg/mL	µM	µg/mL	µM	µg/mL	µM
C1370	25 [B]	21.24 [B]	>50 [ND]	>23.7 [ND]	>50 [ND]	>16.2 [ND]	>50 [ND]	>5.7 [ND]
C1771	25 [B]	21.24 [B]	>50 [ND]	>23.7 [ND]	>50 [ND]	>16.2 [ND]	>50 [ND]	>5.7 [ND]
C1865	12.50 [b]	10.62 [b]	>50 [ND]	>23.7 [ND]	>50 [ND]	>16.2 [ND]	18.75 [B]	2.15 [B]
C3050	25 [b]	21.24 [b]	>50 [ND]	>23.7 [ND]	>50 [ND]	>16.2 [ND]	>50 [ND]	>5.7 [ND]
C1496	18.75 [B]	15.93 [B]	>50 [ND]	>23.7 [ND]	>50 [ND]	>16.2 [ND]	>50 [ND]	>5.7 [ND]
C2613	25 [B]	21.24 [B]	>50 [ND]	>23.7 [ND]	>50 [ND]	>16.2 [ND]	>50 [ND]	>5.7 [ND]
C3698	12.50 [b]	10.62 [b]	>50 [ND]	>23.7 [ND]	>50 [ND]	>16.2 [ND]	>50 [ND]	>5.7 [ND]
C3697	25 [b]	21.24 [b]	>50 [ND]	>23.7 [ND]	>50 [ND]	>16.2 [ND]	37.50 [b]	4.30 [b]
C3699	18.75 [b]	15.93 [b]	>50 [ND]	>23.7 [ND]	>50 [ND]	>16.2 [ND]	9.37 [b]	1.08 [b]
C4750	6.25 [B]	5.31 [B]	0.14 [b]	0.07 [b]	18.75 [B]	6.06 [B]	4.69 [B]	0.54 [B]
X4722	50 [ND]	42.48 [ND]	>50 [ND]	>23.7 [ND]	>50 [ND]	>16.2 [ND]	12.50 [B]	1.43 [B]
X4575	50 [ND]	42.48 [ND]	>50 [ND]	>23.7 [ND]	>50 [ND]	>16.2 [ND]	2.34 [B]	0.27 [B]
X4724	>50 [ND]	>42.5 [ND]	>50 [ND]	>23.7 [ND]	>50 [ND]	>16.2 [ND]	18.75 [b]	2.15 [b]
X4723	25 [ND]	21.24 [ND]	>50 [ND]	>23.7 [ND]	>50 [ND]	>16.2 [ND]	>50 [ND]	>5.7 [ND]
X4725	>50 [ND]	>42.5 [ND]	>50 [ND]	>23.7 [ND]	>50 [ND]	>16.2 [ND]	12.50 [B]	1.43 [B]
X1084	50 [ND]	42.48 [ND]	>50 [ND]	>23.7 [ND]	>50 [ND]	>16.2 [ND]	4.69 [B]	0.54 [B]
X1087	50 [ND]	42.48 [ND]	>50 [ND]	>23.7 [ND]	>50 [ND]	>16.2 [ND]	6.25 [B]	0.72 [B]
C7124	25 [ND]	21.24 [ND]	>50 [ND]	>23.7 [ND]	>50 [ND]	>16.2 [ND]	>50 [ND]	>5.7 [ND]
X1090	25 [ND]	21.24 [ND]	>50 [ND]	>23.7 [ND]	>50 [ND]	>16.2 [ND]	>50 [ND]	>5.7 [ND]
X1097	25 [ND]	21.24 [ND]	>50 [ND]	>23.7 [ND]	>50 [ND]	>16.2 [ND]	>50 [ND]	>5.7 [ND]

Table S8: Microcins McC, MccJ25, MccB17 and MccE492 MIC mean values in µg/mL and µM against *K. pneumoniae* natural isolates. The MBC legend is [b] for bacteriostatic effect, [B] for bactericidal effect and [ND] for an immeasurable MBC, due to no inhibition within the range of microcin concentrations used.

Strain	Microcin							
	McC		MccJ25		MccB17		MccE492	
	µg/mL	µM	µg/mL	µM	µg/mL	µM	µg/mL	µM
C643	9.38 [b]	7.97 [b]	0.09 [B]	0.04 [B]	12.50 [B]	4.04 [B]	9.38 [B]	1.08 [B]
C659	4.69 [b]	3.98 [b]	>50 [ND]	>23.7 [ND]	>50 [ND]	>16.2 [ND]	>50 [ND]	>5.7 [ND]
C660	4.69 [b]	3.98 [b]	>50 [ND]	>23.7 [ND]	>50 [ND]	>16.2 [ND]	>50 [ND]	>5.7 [ND]
C664	6.25 [b]	5.31 [b]	>50 [ND]	>23.7 [ND]	>50 [ND]	>16.2 [ND]	>50 [ND]	>5.7 [ND]
C650	4.69 [b]	3.98 [b]	>50 [ND]	>23.7 [ND]	>50 [ND]	>16.2 [ND]	>50 [ND]	>5.7 [ND]
C651	6.25 [B]	5.31 [B]	>50 [ND]	>23.7 [ND]	>50 [ND]	>16.2 [ND]	>50 [ND]	>5.7 [ND]
C683	9.38 [B]	7.97 [B]	>50 [ND]	>23.7 [ND]	>50 [ND]	>16.2 [ND]	>50 [ND]	>5.7 [ND]
C493	6.25 [b]	5.31 [b]	>50 [ND]	>23.7 [ND]	>50 [ND]	>16.2 [ND]	25.00 [B]	2.87 [B]
AAC 1799	3.13 [b]	2.66 [b]	>50 [ND]	>23.7 [ND]	>50 [ND]	>16.2 [ND]	25.00 [B]	2.87 [B]
AAC 1797	6.25 [b]	5.31 [b]	0.09 [b]	0.04 [b]	12.50 [b]	4.04 [b]	6.25 [B]	0.72 [B]
AAC 1760	6.25 [b]	5.31 [b]	0.09 [b]	0.04 [b]	12.50 [b]	4.04 [b]	9.38 [B]	1.08 [B]
AAC 1791	6.25 [b]	5.31 [b]	0.09 [b]	0.04 [b]	12.50 [b]	4.04 [b]	6.25 [B]	0.72 [B]
AAC 1795	3.13 [b]	2.66 [b]	>50 [ND]	>23.7 [ND]	>50 [ND]	>16.2 [ND]	6.25 [B]	0.72 [B]
1344	6.25 [b]	5.31	>50 [ND]	>23.7 [ND]	>50 [ND]	>16.2 [ND]	2.34 [B]	0.27 [B]

Table S9: Microcins McC, MccJ25, MccB17 and MccE492 MIC mean values in µg/mL and µM for *Salmonella enterica* subsp. *enterica* natural isolates. The MBC legend is [b] for bacteriostatic effect, [B] for bactericidal effect and [ND] for an immeasurable MBC, due to no inhibition within the range of microcin concentrations used.

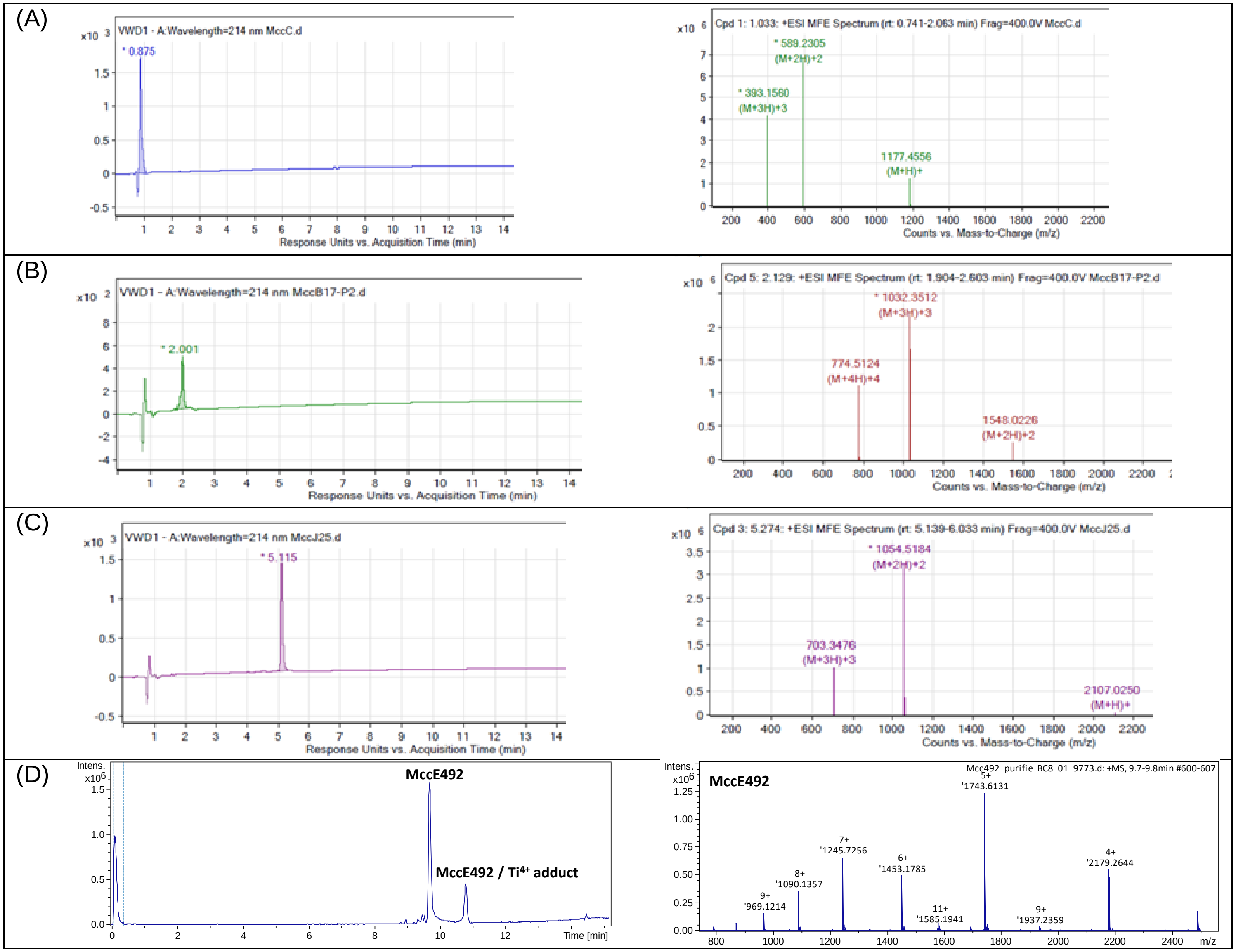

(A)
VWD1 - A:Wavelength=214 nm MccC.d
x10 3
* 0.875
Response Units vs. Acquisition Time (min)
Cpd 1: 1.033: +ESI MFE Spectrum (rt: 0.741-2.063 min) Frag=400.0V MccC.d
x10 6
* 589.2305
(M+2H)+2
* 393.1560
(M+3H)+3
1177.4556
(M+H)+
Counts vs. Mass-to-Charge (m/z)
(B)
VWD1 - A:Wavelength=214 nm MccB17-P2.d
x10 2
* 2.001
Response Units vs. Acquisition Time (min)
Cpd 5: 2.129: +ESI MFE Spectrum (rt: 1.904-2.603 min) Frag=400.0V MccB17-P2.d
x10 6
* 1032.3512
(M+3H)+3
774.5124
(M+4H)+4
1548.0226
(M+2H)+2
Counts vs. Mass-to-Charge (m/z)
(C)
VWD1 - A:Wavelength=214 nm MccJ25.d
x10 3
* 5.115
Response Units vs. Acquisition Time (min)
Cpd 3: 5.274: +ESI MFE Spectrum (rt: 5.139-6.033 min) Frag=400.0V MccJ25.d
x10 6
* 1054.5184
(M+2H)+2
703.3476
(M+3H)+3
2107.0250
(M+H)+
Counts vs. Mass-to-Charge (m/z)
(D)
Intens.
x10^6
MccE492
MccE492 / Ti4+ adduct
Time [min]
Intens.
x10^6
MccE492
Mcc492_purifie_BC8_01_9773.d: +MS, 9.7-9.8min #600-607
5+ 1743.6131
7+ 1245.7256
6+ 1453.1785
8+ 1090.1357
9+ 969.1214
11+ 1585.1941
9+ 1937.2359
4+ 2179.2644
m/z

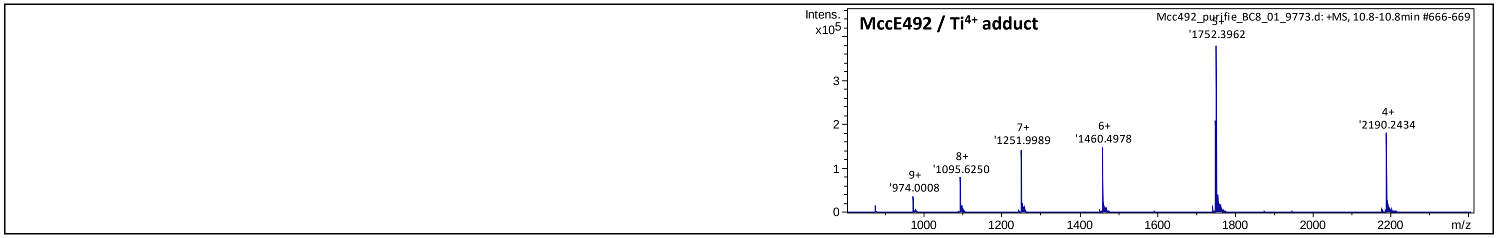

Figure S1: Microcin purification quality control. LC profiles (UV absorbance at 214 nm for A to C, total ion chromatogram for D, on the left) and MS spectra (on the right) of (A) McC, (B) MccB17, (C) MccJ25 and (D) MccE492.

References

1. Metlitskaya, A., et al., *Aspartyl-tRNA synthetase is the target of peptide nucleotide antibiotic Microcin C.* J Biol Chem, 2006. **281**(26): p. 18033-42.
2. Delgado, M.A., et al., *Escherichia coli RNA polymerase is the target of the cyclopeptide antibiotic microcin J25.* J Bacteriol, 2001. **183**(15): p. 4543-50.
3. Li, Y.-M., et al., *From Peptide Precursors to Oxazole and Thiazole-Containing Peptide Antibiotics: Microcin B17 Synthase.* Science, 1996. **274**(5290): p. 1188.
4. Xavier, T., *Siderophore Peptide, a New Type of Post-translationally Modified Antibacterial Peptide with Potent Activity* - Journal of Biological Chemistry.*
5. ATCC, *E. coli 25922*.
6. ATCC. *Salmonella 6962*. Available from: https://www.phe-culturecollections.org.uk/products/bacteria/detail.jsp?refId=NCTC+129&collection=nctc.
